# Accelerated fast spin echo diffusion spectrum imaging in the mouse heart ex-vivo

**DOI:** 10.1186/1532-429X-15-S1-W6

**Published:** 2013-01-30

**Authors:** I Teh, M Lohezic, D Aksentijevic, JE Schneider

**Affiliations:** 1 Department of Cardiovascular Medicine, University of Oxford, Oxford, UK

## Background

Probing the microscale diffusion of water informs on cardiac microstructural features such as cell density and orientation. Diffusion spectrum imaging (DSI) is widely regarded as the gold standard as it measures diffusion in a model-free manner. However, acquisition times are prolonged due to high sampling requirements in q-space. Various methods of reducing acquisition times have been explored, including the use of echo planar imaging [1], and compressed sensing [2,3]. Here we investigate acquiring DSI data using an 8-fold accelerated diffusion-weighted fast spin echo (DW-FSE) pulse sequences versus a diffusion-weighted spin echo (DW-SE) reference.

## Methods

Heart was excised from a C57Bl/6 female mouse (n=1), cannulated via the aorta for a brief Langendorff perfusion with Tyrode solution before high-potassium induced cardioplegic arrest. Karnovsky’s solution was used to fix the tissue for 24hrs, before embedding in 2% agar. MRI was performed using a 9.4T preclinical scanner (Agilent Technologies, Santa Clara, CA), a shielded gradient system (Gmax=1T/m, rise time=130ms, ID=60mm), and an ID=13mm volume Tx/Rx RF coil. 2D multislice data were acquired with: TR=1500ms, Matrix=96x96, FOV=12x12mm, slices=20, thickness=0.5mm, Gmax=900mT/m, δ=2.5ms, Δ=11.26ms, #B0=26, #diffusion encoding vectors=514, Bmax=4000s/mm2. Stejskal-Tanner diffusion gradients were applied about the first refocusing pulse. The DW-SE and DW-FSE data were each acquired twice in succession at 21.6hrs and 2.7hrs per scan. An FA map was generated from the DW-SE data, and thresholded at 0.15 to create a mask that was applied to all data. The diffusion probability density function and orientation distribution function (ODF) were calculated on a voxel-wise basis using Lucy-Richardson deconvolution in Matlab (Mathworks, Natick, MA) [4].

## Results

**Figure 1:**
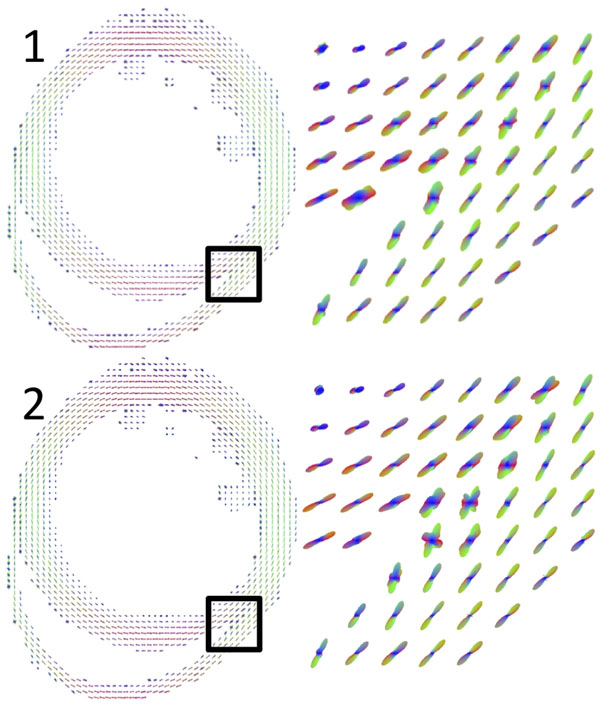
ODFs in the mid-ventricular slice of a mouse heart ex-vivo reconstructed from DW-SE data. (Top and bottom) Two identical scans were acquired. Highlighted regions show the intersection between the right and left ventricles.

**Figure 2:**
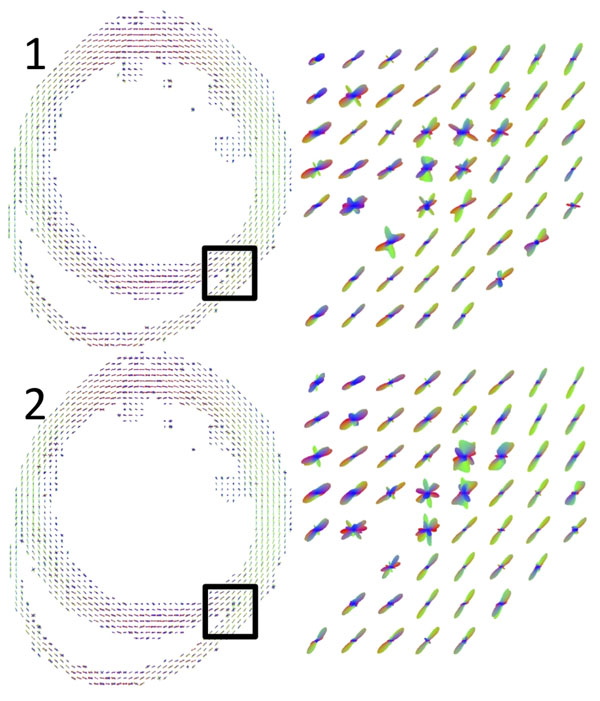
(Top and bottom) ODFs similarly reconstructed from 2 identical sets of DW-FSE data.

Figure 1 depicts the ODFs in a single mid-ventricular slice in the mouse myocardium from two identical DW-SE scans. These facilitate identification of major cell orientations, including the transition in helix angle from the subendocardium to the subepicardium, and the multiple orientations at the intersection of the right and left ventricles. Figure 2 illustrates ODFs similarly reconstructed from DW-FSE data. It shows that the dominant diffusion orientations are preserved, and that there are differences as compared to the DW-SE results, particularly in regions with multiple cell orientations.

## Conclusions

We observed that DW-FSE enables 8-fold acceleration, and provides a visually plausible representation of the ODF. Work is in progress to quantify the precision of the measurements. Future work aims to explore complementary methods to accelerate the acquisition, and to validate the DSI parameters with histology.

## Funding

EPSRC, UK Grant: EP/J013250/1
